# Chinese herbal medicine for recurrent urinary tract infection: formulation-specific clinical evidence, experimental pharmacology, and unresolved recurrence mechanisms

**DOI:** 10.3389/fphar.2026.1950004

**Published:** 2026-10-23

**Authors:** Pengtian Zhang, Xuming Zhang, Chaoyue Ji, Weiguo Hu

**Affiliations:** Beijing Tsinghua Changgung Hospital, Tsinghua University, Beijing, China

**Keywords:** Chinese herbal medicine, ethnopharmacology, microbiome, mucosal immunity, recurrent urinary tract infection, uropathogenic *Escherichia coli*, urothelial barrier

## Abstract

**Ethnopharmacological relevance:**

Chinese herbal medicine (CHM) is used for recurrent or chronic urinary symptoms within the traditional category of dysuria, often through stage-specific treatment during symptomatic and remission phases. Whether defined CHM preparations prevent recurrent bacterial cystitis or modify recurrence biology remains uncertain. The aim of this study is to critically evaluate formulation-specific clinical evidence for CHM in women with recurrent urinary tract infection (rUTI) and determine whether reported benefits are connected to recurrence biology.

**Materials and methods:**

Structured searches of PubMed, China National Knowledge Infrastructure, and Wanfang Data were closed on 13 July 2026. After within-batch screening and cross-source reconciliation, 103 reports were linked into 96 study or unresolved-report clusters. Clusters were classified by directness, and clinical confidence was appraised using allocation, participant flow, recurrence ascertainment, denominator integrity, comparator, intervention identity, safety reporting, internal consistency, and report independence.

**Results:**

Ten core clusters directly addressed prospective recurrence or suppressive treatment, but every comparative estimate was of very low confidence. Reported signals were formulation-specific and were limited by attrition, responder-conditioned denominators, active comparators, co-interventions, poorly defined recurrence, internal numerical inconsistencies, or report linkage. Botanical identity and study-batch chemistry were usually inadequate. Experimental studies supported anti-adhesion, virulence-modulating, anti-inflammatory, immune, and urothelial plausibility, including chemically characterized *Persicaria capitata* extract and more specific Caveolin-1 evidence for purified cynaroside, but predominantly used acute or pretreatment models. No microbiome or biomarker study evaluated clinical mediation.

**Conclusion:**

Current evidence is inconclusive rather than uniformly negative and supports cautious hypothesis generation for specific preparations, not a class-wide efficacy or safety claim. No clinical study demonstrates that CHM reduces recurrence by clearing persistent uropathogens, interrupting gut–bladder or vaginal transmission, restoring microbial ecology, or repairing the urothelial barrier. Reproducible products, intention-to-treat recurrence trials, longitudinal strain-resolved sampling, and prespecified mediation analyses are required.

## 1 Introduction

### 1.1 Ethnopharmacological context and clinical-use rationale

Within Chinese medicine, recurrent or chronic lower urinary symptoms are commonly discussed under the traditional category of dysuria. The modern clinical literature reviewed here operationalizes this tradition through damp-heat-clearing formulae during symptomatic or suppressive phases and deficiency-supporting formulae during remission, including staged Yinqiao Bazheng San/Zhibai Dihuang Tang regimens, syndrome-differentiated Er Ding Er Xian/Zi Shen Qing Li treatment, and Zhuolin granules for liver-depression and damp-heat syndrome (Sheng and Sidike, 2016; Gu et al., 2017; Zhu, 2020; Zou, 2018). These reports document contemporary clinical use but do not establish historical continuity, botanical equivalence, or efficacy. This distinction is central to ethnopharmacological interpretation: a traditional rationale may explain why an intervention was selected, but pharmacological and clinical claims require auditable product identity and outcome evidence.

### 1.2 Clinical heterogeneity and intervention definition

Recurrent urinary tract infection is commonly defined clinically by repeated symptomatic episodes within a specified interval, but the events grouped under this label are not biologically uniform. Recurrence can represent reinfection with a different strain, re-emergence of the same strain from intestinal, vaginal, urinary, intracellular, or tissue-associated reservoirs, or more than one route within the same patient (Ikäheimo et al., 1996; Luo et al., 2012; Mysorekar and Hultgren, 2006; De Nisco et al., 2019; Thänert et al., 2019; 2022). Current prevention strategies include behavioral measures, vaginal estrogen in appropriate postmenopausal women, selected non-antibiotic options, and continuous, post-coital, or patient-initiated antimicrobial strategies (Anger et al., 2025; European Association of Urology, 2026). Persistent interest in botanical and traditional approaches reflects concerns about antimicrobial exposure, resistance, tolerability, and incomplete prevention, but therapeutic need does not substitute for reliable evidence.

Chinese herbal medicine (CHM) is not one pharmacological intervention. The clinical literature includes commercial Chinese polyherbal preparations (CCPPs) such as Sanjin tablets, hospital empirical preparations, fixed or modified classical-formula preparations, individualized prescriptions based on syndrome differentiation, and combinations with antibiotics, estrogen, moxibustion, acupuncture, or external therapy. These categories describe how the test material was supplied and individualized; they do not establish comparable regulation, chemistry, or efficacy. The interventions differ in botanical drugs, dose, preparation, manufacturer, treatment phase, comparator, and therapeutic logic. A result for one formulation therefore cannot be generalized automatically to CHM as a class.

### 1.3 Aim and linked review questions

Mechanistic overreach is a second problem. Contemporary recurrent urinary tract infection (rUTI) biology emphasizes intracellular bacterial communities, quiescent reservoirs, bladder-wall bacteria, gut and urogenital ecology, and persistent immune or epithelial states after infection (Mysorekar and Hultgren, 2006; De Nisco et al., 2019; Worby et al., 2022; Wu et al., 2020; Russell et al., 2023). CHM studies often report antibacterial activity, cytokine changes, immune indices, or altered microbial taxa, but these observations rarely connect prospectively to recurrent bacterial cystitis. This review therefore asks two linked questions: which defined CHM interventions have produced auditable clinical signals in women with recurrent bacterial lower UTI, and has any reported benefit been connected causally to pathogen persistence, transmission, microbial ecology, mucosal immunity, or urothelial barrier biology?

## 2 Materials and methods

### 2.1 Review design, population, and terminology

This article is a critical review informed by a structured search and report-level evidence mapping. It was not designed or reported as a systematic review and does not claim exhaustive retrieval of every Chinese-language record. The primary population comprised nonpregnant adult women with recurrent localized bacterial cystitis and without major urinary tract abnormalities, indwelling catheters, systemic infection, transplant status, or major immunosuppression. Studies in men, children, patients with structural disease or major metabolic comorbidity, and acute UTI without a recurrent phenotype were used only as supporting or peripheral evidence.

Following current EAU and AUA/CUA/SUFU guidance, guideline-defined recurrent cystitis was operationalized as at least two separate symptomatic episodes of acute bacterial cystitis within 6 months or at least three within 12 months. Recurrent bacterial cystitis is reserved for localized episodes supported by urine testing or culture when reported. When a primary report did not provide sufficient episode-level information to verify this threshold, its original terminology was retained as source-reported, and the population was not automatically classified as guideline-defined rUTI. “Chronic UTI” is retained only when it was the source report’s terminology and is not assumed to satisfy a modern rUTI definition. Relapse or same-strain persistence requires strain-level or otherwise convincing linkage; reinfection refers to a distinct infecting strain. Composite “total effectiveness” categories were not treated as recurrence outcomes. Recurrence outcomes were ranked by whether they were prospective, symptom-defined, culture-confirmed, and analyzed in all randomized participants rather than in completers or treatment responders.

### 2.2 Information sources and search closure

PubMed, China National Knowledge Infrastructure (CNKI), and Wanfang Data were searched from database inception to 13 July 2026. The executed PubMed query combined controlled vocabulary and title/abstract terms for recurrent UTI or cystitis with Chinese medicine, botanical preparations, named formulae, and dosage forms. It contained no date, language, or human-study filter and yielded 179 unique PMIDs. Broad Chinese-language searches yielded 42 CNKI and 28 Wanfang records. Because those searches missed known formula-specific reports, three prespecified named-formula groups (NF-A–NF-C) were run in both Chinese databases using the same recurrent/chronic-UTI disease block and different formula blocks.

Each database batch was exported and screened separately before cross-database report linkage. Platform carry-over was retained in the audit trail but not counted as a new batch-attributable record: 20 of 39 Wanfang NF-B rows were carried over from NF-A, and 39 of 59 Wanfang NF-C rows were carried over from earlier batches. An invalid CNKI NF-C export that repeated NF-B was not counted; the corrected NF-C export contained 37 records. Search-batch yields are therefore not summed as if they were unique records. The SinoMed/Chinese Biomedical Literature Database could not be searched because institutional access was unavailable; this was treated as an access limitation, not as a zero-result search. After the prespecified searches were completed, the discovery set was frozen. Subsequent work was restricted to targeted full-text retrieval, report linkage, denominator adjudication, bibliographic verification, and reference audit. Complete executed search strings, batch yields, and handling of carry-over records are provided in Supplementary Table S3, and the search and evidence-mapping flow is shown in Figure 1.

**FIGURE 1:**
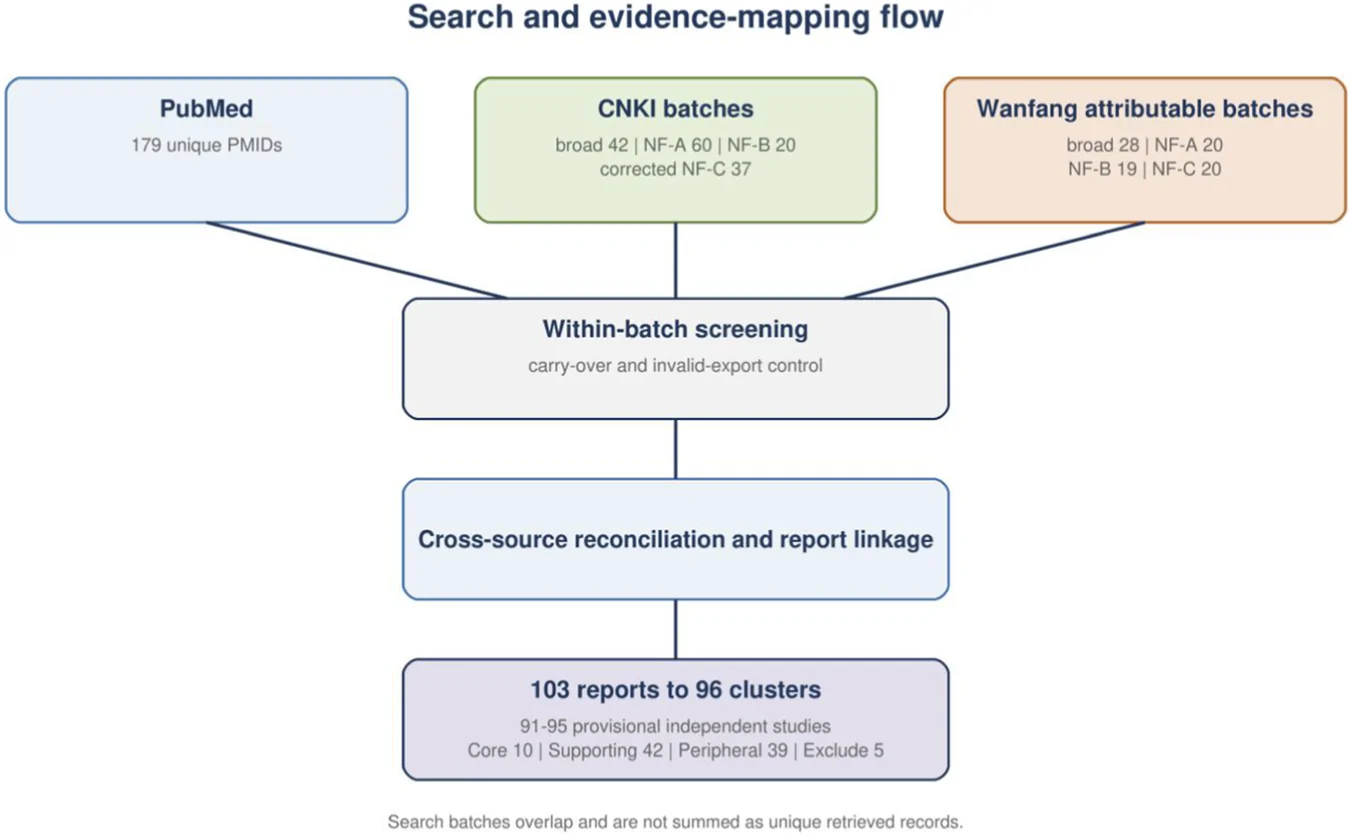
Evidence-labeled biological pathways relevant to recurrent UTI and hypothesized CHM intervention points. Navy links indicate relations supported within the labeled evidence domain. Orange links indicate hypothesized CHM effects for which clinical mediation has not been demonstrated.

### 2.3 Report linkage, directness hierarchy, and confidence appraisal

Reports were linked into study clusters using author group, institution, recruitment period, sample size, intervention, comparator, treatment duration, follow-up, outcomes, and publication chronology. Clinical articles, theses, conference reports, biomarker papers, and microbiome substudies were counted once when they represented the same cohort. Suspected derivative or near-copy publications were not accepted as independent replication without auditable evidence of distinct recruitment. Key report-linkage and research-integrity adjudications are summarized in Supplementary Table S2.

Clusters were assigned to four interpretive levels. Core evidence required an adult female recurrent- or chronic-UTI population, a prospectively assigned CHM intervention, and an extractable prospective recurrence or suppressive-treatment outcome. Supporting evidence retained at least one clinically relevant link but failed one core requirement because of a mixed or indirect population, no extractable participant-level recurrence denominator, inseparable multimodal treatment, a feasibility design, or unavailable full methods. Peripheral evidence was uncontrolled, phenotype-uncertain, comorbid, title-only, mechanistically indirect, or lacked a recurrence-relevant clinical link. Excluded records were outside CHM scope, non-study material, or internally unusable. Reporting defects alone did not determine the core/supporting/peripheral label; the hierarchy primarily represents directness and extractability. Core status therefore denoted directness to the question, not methodological quality.

Confidence was judged narratively rather than by a formal GRADE procedure. The principal domains were allocation and concealment, blinding, participant flow and analysis population, prospective definition and confirmation of recurrence, denominator integrity, comparator and co-intervention, consistency of statistical claims with reported counts, linked or derivative publication, safety ascertainment, and reproducibility of the botanical intervention. A cluster was described as very low confidence when one domain was critically compromised or when at least two domains had serious limitations capable of changing the direction or magnitude of the apparent effect. No core cluster escaped both conditions. “Very low confidence” is therefore an operational interpretive description used in this review, not a formal GRADE certainty rating. The study-level judgments and reasons are reported in Supplementary Table S1.

### 2.4 Data use and synthesis

Effects were synthesized narratively by formulation and independent study cluster. Numerical recurrence results were retained only when events and denominators were identifiable. Responder-conditioned estimates, unsupported P-values, abstract-only effect sizes, and inseparable multimodal effects were not treated as reliable comparative estimates. No quantitative pooling was undertaken because formulations, comparators, co-interventions, recurrence definitions, treatment phases, follow-up, and reporting quality were too heterogeneous. Table 1 summarizes all core clusters, and Supplementary Table S1 provides the full cluster-level matrix of 10 core and 42 supporting clusters.

**TABLE 1:** Core clinical study clusters. Core indicates direct relevance to the target question, not methodological quality. Confidence descriptions are narrative and non-GRADE.

Intervention/report	Design and population	Treatment/follow-up	Reported recurrence signal	Denominator/outcome issue	Principal limitations and interpretation
Modified Bazheng formula (Liu et al., 2019)	Double-blind, double-dummy trial; 122 women	4 weeks/6 months	5/61 vs 7/61 reported; no significant difference	Only 48/50 remained in recurrence analysis; recovered-participant outcome; inconsistent missing-data assumptions	No prespecified non-inferiority margin or CI; no valid non-inferiority claim
Individualized CHM (Gu et al., 2024)	Multicenter randomized study; 300 enrolled, 230 completers	12 weeks/3 months	18/114 vs 31/116 during treatment; 11/96 vs 36/85 thereafter	Completer and responder-conditioned analyses; disposition of 70 participants insufficiently reported	Active one-tenth-dose herbal comparator; formula-specific, very low-confidence signal
Sanjin/antibiotic/combination (Lü et al., 2008)	Three-arm study; 95 women in remission	6 months/no separate follow-up	12/32, 13/31, and 4/32	Participant-level 6-month recurrence	Sanjin alone not superior; combination is an adjunctive signal; small open study
Zhuolin program (Zhu et al., 2019 ; 2022; Zhu, 2020)	120 postmenopausal women; linked thesis, clinical, and microbiome reports	14 weeks/6 months	5 reported recurrences vs 28	Rates calculated after losses and among post-randomization responders	Clinical signal with the responder denominator; microbiome substudy did not test mediation
Sanjin maintenance (Yin et al., 2011)	Open randomized study; article states 68, groups total 58	3 months/3 months off treatment	Percentages imply 2/30 vs 4/28	Unexplained participant-count discrepancy	Reported P < 0.01 unsupported; quantitative recurrence effect unusable
Staged Yinqiao Bazheng → Zhibai Dihuang (Sheng and Sidike, 2016)	Odd/even allocation; 54 in body, 51 in abstract	Approx. 6 weeks/6 months	0/27 vs 4/27	Undefined recurrence; exact two-sided P ≈ 0.111	Predictable allocation and inconsistent sample size; effect not robust
Staged Yinqiao Bazheng → Zhibai Dihuang (Zou, 2018)	Nonrandom treatment-plan comparison; 92 women	Staged regimen/6 months	0/46 vs 6/46	Undefined recurrence and follow-up completeness	Confounded, nonrandom, protocol-specific signal
Modified Gualou Qumai Wan (Guo and Wang, 2026)	Coin-toss allocated adjunctive study; 80 perimenopausal women	10 days/3 and 6 months	2/40 vs 9/40	Recurrence not clearly culture confirmed	Dual intravenous antibiotic co-treatment; adjunctive formula-specific signal
Er Ding Er Xian/Zi Shen Qing Li (Gu et al., 2017)	102 randomized; 93 completers	12 weeks/3 months	5/50 vs 9/43 during treatment; 3/45 vs 13/34 thereafter	Completer- and responder-conditioned denominators	Active one-tenth-dose herbal comparator; very low-confidence signal
Fuzheng Qingre Lishi (Gao et al., 2025)	68 screened; 66 randomized; 62 analyzed	4 weeks/6 months	4/31 vs 11/31	Participant flow supports 31/31, but some efficacy percentages imply 32/group	Incomplete formula and very small mechanistic subsets; signal only, no causal mechanism

## 3 Clinical evidence

### 3.1 Evidence landscape and previous syntheses

After within-batch screening, cross-database reconciliation, and report linkage, the frozen inventory contained 103 report records mapped to 96 study or unresolved-report clusters and an estimated 91–95 provisional independent studies. Ten clusters were classified as core, 42 as supporting, 39 as peripheral, and five as exclude. These counts describe the breadth of the mapped literature, not the number of reliable randomized trials. Multiple programs generated linked clinical, mechanistic, thesis, conference, or microbiome reports, and report-level counts would therefore overstate replication.

A 2024 meta-analysis included 22 randomized trials and 1,934 participants and reported adjunctive signals during treatment and follow-up (Hou et al., 2024). The interventions and populations were heterogeneous, analyses were largely per protocol, and the review did not provide a study-level risk-of-bias synthesis or GRADE assessment. The earlier Cochrane review included seven trials and 542 women, judged all trials to be at high risk of bias, and rated the evidence as being of very low quality (Flower et al., 2015). The compatible conclusion is not established class-wide efficacy but the existence of formula-specific signals in a clinically and methodologically fragile literature.

### 3.2 Better-described randomized and feasibility studies

A multicenter study of individualized traditional Chinese medicine reported that 300 eligible women were enrolled and underwent blinded 1:1 allocation, but only 230 completers (114 and 116) appeared in the efficacy analyses (Gu et al., 2024). The report did not provide a sufficiently transparent arm-specific disposition for the other 70 participants. It is relevant because it evaluated two syndrome-differentiated base prescriptions during remission and prospectively monitored urine testing. However, the completer analysis, unclear allocation concealment, a one-tenth-dose herbal comparator not shown to be pharmacologically inert, and participant-specific modifications without ingredient-frequency reporting materially limit inference. Broad laboratory safety monitoring was described, but arm-level adverse-event and laboratory-abnormality results were not reported.

The modified Bazheng trial used a randomized double-blind double-dummy design and was among the better-described formulation studies (Liu et al., 2019). The report stated 5/61 versus 7/61 recurrences in its intention-to-treat presentation, although only 48 and 50 participants, respectively, remained in the 6-month recurrence analysis, and the calculation was further restricted to recovered participants. Between-group differences were not statistically significant, and the study did not prespecify a non-inferiority margin or demonstrate non-inferiority using confidence intervals. Missing participants were treated inconsistently across the reported analytic approaches, including an analysis that counted missing participants as cured. The study therefore cannot support a valid claim of non-inferiority to antibiotic prophylaxis, even though the fixed formula, manufacturer, and batch were comparatively well described.

The Tailin formulation was evaluated as an adjunct to continuous low-dose antibiotics (Li et al., 2022). The final analysis included 195 participants, of whom only 134 were women (68/98 and 66/97), so the result cannot be treated as a female-only estimate. Reported recurrence at 12 weeks after treatment was 19/98 with Tailin plus rotating low-dose antibiotics and 46/97 with antibiotics plus placebo. The publication described blood counts, liver and kidney function, electrocardiography, and serious-event monitoring but reported safety only as an absence of significant changes or obvious adverse reactions. Registry and publication documents were also inconsistent regarding the identifier and primary outcome. A linked THP/PTX3 report derives from the same cohort and is not an independent efficacy study. The biomarker results are exploratory and do not validate prediction or mediation. The later Tailin Fang II mouse preparation also cannot be assumed to be chemically or compositionally equivalent to the four-drug clinical Tailin formula (Li et al., 2024).

The UK RUTI feasibility trial showed that standardized and individualized CHM could be manufactured and delivered within a regulated primary-care trial framework, but it was not powered to provide reliable efficacy evidence (Flower et al., 2019). The standardized products were manufactured through a documented supply chain, with regulatory review, recognized good-manufacturing-practice certification, marker-compound testing, contaminant testing, and stability assessment. Recruitment and retention were difficult, and the individualized comparison was invalidated in its first phase when prescriptions intended as placebo were found to contain approximately 70%–80% active botanical drugs. Its main contribution is therefore feasibility, product-quality, safety-monitoring, and trial-design learning rather than an efficacy estimate.

### 3.3 Sanjin tablets

The most directly extractable Sanjin recurrence data were obtained from a 2008 three-arm study on 95 women in remission (Lü et al., 2008). During 6 months of suppressive treatment, recurrence occurred in 12/32 participants receiving Sanjin, 13/31 receiving rotating low-dose antibiotics, and 4/32 receiving the combination. Sanjin alone did not outperform antibiotic suppression. The lower recurrence in the combination arm is an adjunctive signal, but sequence generation, concealment, blinding, and strain-level classification were not reported.

A 2009 study randomized 60 women to Sanjin or rotating antibiotics and included 10 healthy controls (Xu and Chen, 2009). “Recent cure” was reported in 18/30 versus 11/30, and composite total effectiveness was reported in 28/30 versus 22/30. Although recent cures were intended to undergo monthly urine testing for 6 months, arm-specific 6-month recurrence events were not reported. T-cell subsets and urinary secretory IgA changed with Sanjin, but no mediation analysis connected these changes to recurrence.

A 2011 open randomized study compared antibiotics plus Sanjin followed by Sanjin maintenance with antibiotics followed by low-dose antibiotic maintenance (Yin et al., 2011). The article repeatedly stated that 68 participants were enrolled, whereas the reported groups contained 30 and 28 participants. Post-discontinuation percentages imply 2/30 versus 4/28 recurrences. The reported P < 0.01 is incompatible with these counts; recalculation is not significant. This study is directly relevant but cannot support a quantitative recurrence claim.

### 3.4 Er Ding Er Xian, Zi Shen Qing Li, and publication integrity

A 2017 study randomized 102 women to syndrome-differentiated Er Ding Er Xian or Zi Shen Qing Li treatment versus rotating low-dose antibiotics plus a one-tenth-dose herbal preparation described as placebo (Gu et al., 2017). Ninety-three participants completed 12 weeks. Recurrence during treatment was 5/50 versus 9/43. Post-treatment recurrence was reported as 3/45 versus 13/34 among participants who had remained recurrence-free during treatment, creating a responder-conditioned denominator rather than an intention-to-treat estimate. Because pharmacological inactivity of the low-dose preparation was not established, this comparator further complicates interpretation.

A 2019 paper reproduced the title, protocol structure, statistical language, discussion, and references of the 2017 report while changing authors, institutions, dates, and total sample and omitting auditable group-level recurrence data. Because substantial textual and structural overlap was accompanied by no auditable evidence of distinct recruitment, it was not counted as an independent replication. A separate 2020 report was not the same cohort, but its abstract and methods described incompatible interventions, and its significance claim was not supported by the reported cure counts; it was excluded from efficacy synthesis.

### 3.5 Zhuolin granules and other direct signals

The Zhuolin program included a doctoral thesis, a clinical article, and a linked gut-microbiome paper (Zhu et al., 2019; 2022; Zhu, 2020). These reports represent one study program. The clinical study suggested fewer recurrences, but recurrence was calculated among post-randomization responders rather than all randomized participants. The microbiome substudy was very small, with only five and four post-treatment stool samples in the randomized groups, and it did not test mediation, strain-level transmission, negative controls, or multiplicity-adjusted associations.

Fuzheng Qingre Lishi therapy reported 4/31 versus 11/31 recurrences, but the parent report stated one sample size, while its outcome denominators implied another, and microscopy, immune, iron-metabolism, and proteomic analyses were exploratory, with some assays using only three samples per group (Gao et al., 2025). Modified Gualou Qumai Wan was associated with 2/40 versus 9/40 reported recurrences in a coin-toss allocated perimenopausal study, but both groups received intensive antibiotic treatment, and culture-defined recurrence was not established (Guo and Wang, 2026). These are adjunctive, formulation-specific signals rather than evidence for CHM monotherapy or a class effect.

### 3.6 Staged Yinqiao Bazheng and Zhibai Dihuang regimens

Two direct reports evaluated a closely similar staged regimen: antibiotics plus modified Yinqiao Bazheng San during acute treatment, followed by modified Zhibai Dihuang Tang after antibiotic withdrawal. Both articles provided base ingredients, doses, symptom-based additions, and alternate-day administration, but neither reported herbal sourcing, batch information, botanical authentication, chemical characterization, a reproducible recurrence definition, or structured safety ascertainment (Sheng and Sidike, 2016; Zou, 2018).

The 2016 report used visit-order odd/even assignment. Its abstract stated 51 participants, whereas the methods and tables used 54 participants, 27 per group. Six-month recurrence was reported as 0/27 versus 4/27. A two-sided Fisher exact test yields P ≈ 0.111, and a continuity-corrected chi-square test yields P ≈ 0.119; the stated P < 0.05 is unsupported by these reported counts under either test. The 2018 report allocated 92 women according to the treatment plan rather than randomization and reported 0/46 versus 6/46 recurrences. Although the event difference is compatible with conventional significance testing, the design is confounded and the outcome is undefined. Different authors, institutions, recruitment periods, and sample sizes make separate recruitment plausible, so the reports are retained as two provisional studies. Their nearly identical protocols, however, mean that they should be described as two methodologically weak reports of one staged therapeutic strategy, not as independent replication of efficacy.

### 3.7 Safety and clinical interpretation

Safety reporting was inconsistent and usually underdeveloped. The Sanjin three-arm study monitored blood counts, liver enzymes, renal function, and electrocardiography; it reported three mild aminotransferase elevations in the antibiotic-only arm and none in the Sanjin-containing arms but did not provide a detailed causality or interaction assessment. The Zhuolin trial reported gastrointestinal discomfort in 2/60 participants receiving the botanical preparation versus 6/60 in the antibiotic arm, with one liver-enzyme elevation and five episodes of leukopenia in the antibiotic arm. The modified Bazheng trial measured alanine aminotransferase, aspartate aminotransferase, and creatinine but reported only that no significant adverse effects occurred. The UK feasibility trial prospectively measured alanine aminotransferase and creatinine and described withdrawals for persistent nausea and severe gastric pain/acute gastritis, but its small size precludes estimation of uncommon harms. The individualized 2024 study specified extensive laboratory surveillance, whereas its results did not provide arm-level adverse-event counts. The Tailin trial likewise reported no significant laboratory changes or obvious adverse reactions without presenting event-level data.

These observations demonstrate variable reporting intensity, not comparative safety or absence of risk. None of the small studies was powered for uncommon or delayed toxicity, and most did not report adherence-adjusted exposure, predefined causality, concomitant non-study products, or systematic herb–drug interaction assessment. In trials using low-dose herbal preparations as putative placebos, pharmacological inactivity was not demonstrated; possible residual activity complicates adverse-event comparisons, although the direction of any resulting bias remains unknown. Formulae varied substantially in botanical drugs, processing, dose, and co-administered antibiotics; safety findings therefore cannot be transferred across preparations. Preparations containing processed aconite, such as modified Gualou Qumai Wan, require product-specific identity, processing, dose, and cardiac–neurological safety interpretation rather than reassurance based on other CHM studies.

Across the core clusters, the permitted interpretations differ by treatment role. For botanical monotherapy or substitution, modified Bazheng did not show a statistically significant difference from its antibiotic strategy and lacked a valid non-inferiority analysis, while Sanjin alone did not outperform rotating antibiotic suppression. For adjunctive treatment, the Sanjin combination arm, Tailin plus low-dose antibiotics, staged botanical–antibiotic regimens, and modified Gualou Qumai Wan generated signals, but each botanical contribution was inseparable from co-treatment or affected by population and design limitations. Individualized CHM, Er Ding Er Xian/Zi Shen Qing Li, Zhuolin, and Fuzheng Qingre Lishi reported favorable recurrence patterns, but active comparators, attrition, responder-conditioned analyses, incomplete product identity, or internal inconsistencies prevented reliable effect estimation. Several other clinically relevant studies did not report an extractable recurrence denominator.

The most defensible clinical conclusion is therefore not that all tested interventions were ineffective, but that the current reports do not establish a causal preventive effect. The signals are formulation- and setting-specific, and confidence in every comparative core estimate is very low. They cannot be generalized to CHM as a class.

## 4 Recurrence biology and microbial ecology

### 4.1 Persistence, reinfection, and intracellular reservoirs

Human recurrence studies show that same-strain persistence and different-strain reinfection both occur (Ikäheimo et al., 1996; Luo et al., 2012). Strain-resolved longitudinal work further indicates heterogeneous routes that can include intestinal persistence, dual gut–urinary persistence, and urinary persistence (Thänert et al., 2019; 2022). These studies establish heterogeneity rather than one universal reservoir model.

Uropathogenic strains also differ in virulence repertoires. Type 1-pilus FimH and P-pilus PapG contribute to adhesion and tissue tropism, flagellin-related motility can facilitate access to or movement within the urinary tract, and toxins, iron-acquisition systems, capsules, and biofilm-associated phenotypes can alter experimental fitness. The cynaroside study directly measured changes in *fimH*, *papG*, and *fliC* and related adhesion and invasion phenotypes (Zheng et al., 2026). However, none of the CHM clinical studies performed longitudinal strain-resolved virulence profiling, and a change in one acute experimental virulence readout is neither a validated predictor of human recurrence nor evidence of reservoir eradication.

In mice, uropathogenic *E. coli* can form intracellular bacterial communities and quiescent intracellular reservoirs that survive apparent urine sterilization and later re-emerge (Mysorekar and Hultgren, 2006). IBC-like cells and filamentous bacteria have also been detected in urine from a subset of women with acute cystitis (Rosen et al., 2007). Bladder-wall bacteria and chronic inflammatory changes were visualized in a highly selected cohort of postmenopausal women with refractory rUTI (De Nisco et al., 2019). Transient urogenital microbial exposure can reactivate dormant *Escherichia coli* and worsen recurrent disease in mice (Gilbert et al., 2017). These observations support biological plausibility but do not prove that every human recurrence arises from an intracellular or tissue reservoir.

### 4.2 Gut–bladder and vaginal–bladder ecology

The gut is a plausible source of uropathogens. In selected longitudinal cohorts, intestinal abundance or persistent carriage of uropathogenic strains preceded or accompanied urinary events (Magruder et al., 2019; Thänert et al., 2019; 2022). Year-long multi-omics sampling in women with a history of rUTI identified gut ecological and immune differences, while UTI-associated strains often persisted in the gut despite antibiotics (Worby et al., 2022). These associations do not establish direct transfer during every episode and remain vulnerable to antibiotic, behavioral, and host confounding.

The bladder and vagina also form an interconnected urogenital ecology. Catheterized bladder and paired vaginal isolates can be closely related at the genome level (Thomas-White et al., 2018), and UPEC can invade vaginal epithelial cells in experimental systems and mouse models (Brannon et al., 2020). Human evidence for a durable intracellular vaginal reservoir remains preliminary. Cross-sectional urinary or urogenital microbiome studies caution against a simple universal “Lactobacillus-depleted dysbiosis” model: active infection is often uropathogen-dominated, but asymptomatic rUTI and control groups can overlap, and prophylactic antibiotics themselves shape observed communities (Vaughan et al., 2021; Neugent et al., 2022).

### 4.3 Post-infection immune and epithelial states

Repeated infection can alter the response to subsequent challenge. Mouse studies show that a Th2-skewed bladder response may favor epithelial repair at the expense of protective clearance after reinfection (Wu et al., 2020), while infection history can imprint persistent urothelial stem-cell states that modify later susceptibility or resistance (Russell et al., 2023). These models provide causal experimental frameworks but have not been validated as uniform mechanisms in women with rUTI. The recurrence pathways and hypothesized CHM intervention points are summarized in Figure 2.

**FIGURE 2:**
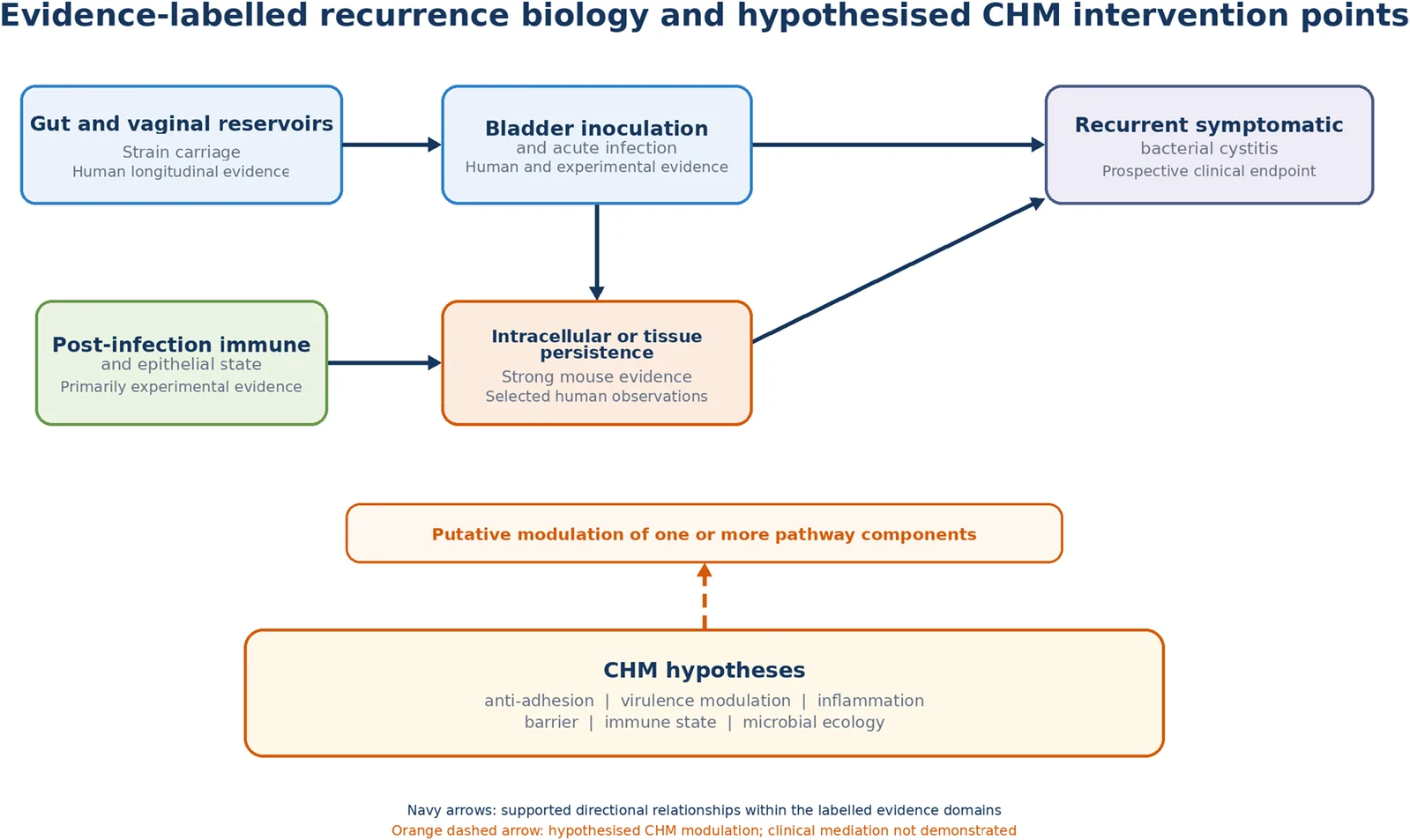
Non-linear evidence requirements for demonstrated clinical mediation. Material identity, preclinical causality, human intermediate biomarkers, and valid clinical recurrence-effect estimation are complementary domains. Evidence in one domain does not automatically establish another, and no current CHM study demonstrates clinical mediation.

## 5 Experimental pharmacology of CHM

### 5.1 Pathogen-directed effects

Experimental studies report effects on bacterial growth, motility, adhesion, invasion, biofilm-associated behavior, extracellular-vesicle signaling, and virulence-related phenotypes. The aqueous *Persicaria capitata* (Buch.-Ham. ex D. Don) H. Gross extract study provided comparatively strong chemical characterization: a total of 78 constituents were tentatively profiled by high-resolution mass spectrometry, and gallic acid, protocatechuic acid, quercitrin, and quercetin were quantified using a validated UPLC–MS/MS method (Zhou et al., 2025). The extract did not directly inhibit UPEC growth at the tested concentrations while altering motility and fimbrial/flagellar structure and reducing adhesion or invasion in T24 cells. These experiments used concentrations from 50 to 200 μg/mL for cell assays and an acute rat model with 0.32–1.28 g/kg/day; oral dosing began 2 days before a single intravesical challenge. The chemical analysis is a positive reporting example, whereas the pretreatment design and absence of a relapse phase limit recurrence inference.

Cynaroside, a purified flavonoid rather than a botanical preparation, similarly showed no direct bacteriostasis but reduced UPEC motility, biofilm-associated phenotypes, fimbrial/flagellar structures, and expression of *fimH*, *papG*, and *fliC* (Zheng et al., 2026). The experiments used 125–500 μg/mL in several bacterial assays, 10–40 μM in 5,637 urothelial cells, and 10–40 mg/kg/day in an acute rat model in which dosing also began 2 days before infection. Modified Zhibai Dihuang preparations have additionally been studied against extended-spectrum beta-lactamase-producing *E. coli* in a rat UTI model, with effects on bacterial burden and biofilm-associated readouts (Chen et al., 2023). These findings support acute pharmacological plausibility, but they do not demonstrate clearance of quiescent reservoirs, eradication of tissue-associated bacteria after antibiotic treatment, or prevention of spontaneous relapse.

### 5.2 Host-directed and urothelial effects

Clinical and preclinical reports describe changes in inflammatory mediators, immune-cell subsets, serum immunoglobulins, urinary secretory IgA, Tamm–Horsfall protein, pentraxin 3, estrogen-related markers, iron metabolism, and urothelial injury. In the Yinhua Miyanling Tablet study, 10 women with uncomplicated UTI or an acute episode in the setting of recurrent UTI received the product for 28 days without a concurrent control; urine microscopy, inflammatory markers, blood transcriptomics, and urine metabolomics were measured before and after treatment (Zheng et al., 2025). A separate single-challenge mouse experiment used 0.5–2 g/kg/day and supported modulation of the TLR4/MyD88/p38 MAPK/NFκB pathway. The study therefore links a defined finished-product batch to human intermediate biomarkers and acute experimental pharmacology, but not to a comparative recurrence outcome or a clinical mediation analysis.

The cynaroside study provides more specific preclinical evidence for a urothelial pathway: *CAV1* knockdown attenuated its anti-adhesion and barrier effects, whereas expression of an siRNA-resistant *CAV1* construct partially restored them (Zheng et al., 2026). This perturbation-and-rescue design strengthens causal interpretation within the cell model, but the same pathway has not been shown to mediate recurrent cystitis in treated patients. Tailin Fang II experiments integrate innate immune and bladder-injury pathways (Li et al., 2024). However, most other biomarker studies are small, secondary, unblinded, and disconnected from validated recurrence endpoints. A post-treatment biomarker difference is not evidence that the marker lies on the causal pathway to fewer infections, and related formula names do not establish that an experimental preparation matches a clinical intervention.

### 5.3 Microbiome evidence

Only the Zhuolin program directly paired a CHM clinical study with gut-microbiome analysis in women with rUTI (Zhu et al., 2019; 2022; Zhu, 2020). The substudy was underpowered, experienced severe post-treatment sample loss, and did not use strain-resolved tracking, participant-level recurrence–microbiome linkage, or a prespecified mediation model. The cynaroside study also used metagenomic sequencing of clean-catch urine from 14 women with UTI and 13 healthy controls to generate mechanistic hypotheses, but this was a small cross-sectional comparison rather than a longitudinal treatment analysis (Zheng et al., 2026).

Urine is a low-biomass specimen in which reagent contamination, collection method, microbial load, and sequencing batch can materially influence apparent community differences. The main reports did not document a complete control framework including extraction and reagent blanks, positive controls or mock communities, microbial biomass quantification, randomized laboratory batches, and prespecified contaminant removal. Recent antibiotic exposure can also alter both pathogen burden and community composition and should be treated as a time-varying confounder rather than a background characteristic. Taxonomic change or cross-sectional separation therefore cannot establish interruption of gut–bladder transmission or microbiome-mediated recurrence prevention.

### 5.4 Model taxonomy and translational limits

Most CHM experiments used a single acute inoculation, pretreatment before infection, short observation, or non-recurrent inflammatory models. The *P. capitata* and cynaroside rat studies started dosing before the single UPEC challenge; the Yinhua Miyanling Tablet mouse experiment evaluated short-term treatment of one acute infection. None incorporated antibiotic clearance followed by spontaneous relapse, strain-resolved gut-to-bladder reseeding, or natural reactivation of a quiescent reservoir. Repeated transurethral challenge is more relevant than a single inoculation but still does not reproduce all of these processes. Causal rescue experiments were present in the cynaroside cell study but were otherwise uncommon; microbiota-transfer studies, antibiotic-treatment and relapse phases, and blinded outcome assessment were generally absent. Model labels should therefore describe what was performed rather than implying a true recurrent-UTI phenotype.

## 6 Discussion

### 6.1 The unresolved mechanism–clinical evidence gap

The central gap is not whether CHM can change bacteria, inflammatory markers, immune indices, or microbial taxa. It is whether a defined biological change mediates a reduction in prospectively confirmed recurrent bacterial cystitis. No study combined intention-to-treat recurrence outcomes with longitudinal stool, vaginal, catheterized urine, and urothelial sampling; strain-resolved genomics; and prespecified causal mediation. Figure 3 separates biological plausibility, observed intermediate biomarkers, and the evidence required to establish clinical mediation. None of the measured biomarkers has been validated as a surrogate endpoint for recurrence.

**FIGURE 3:**
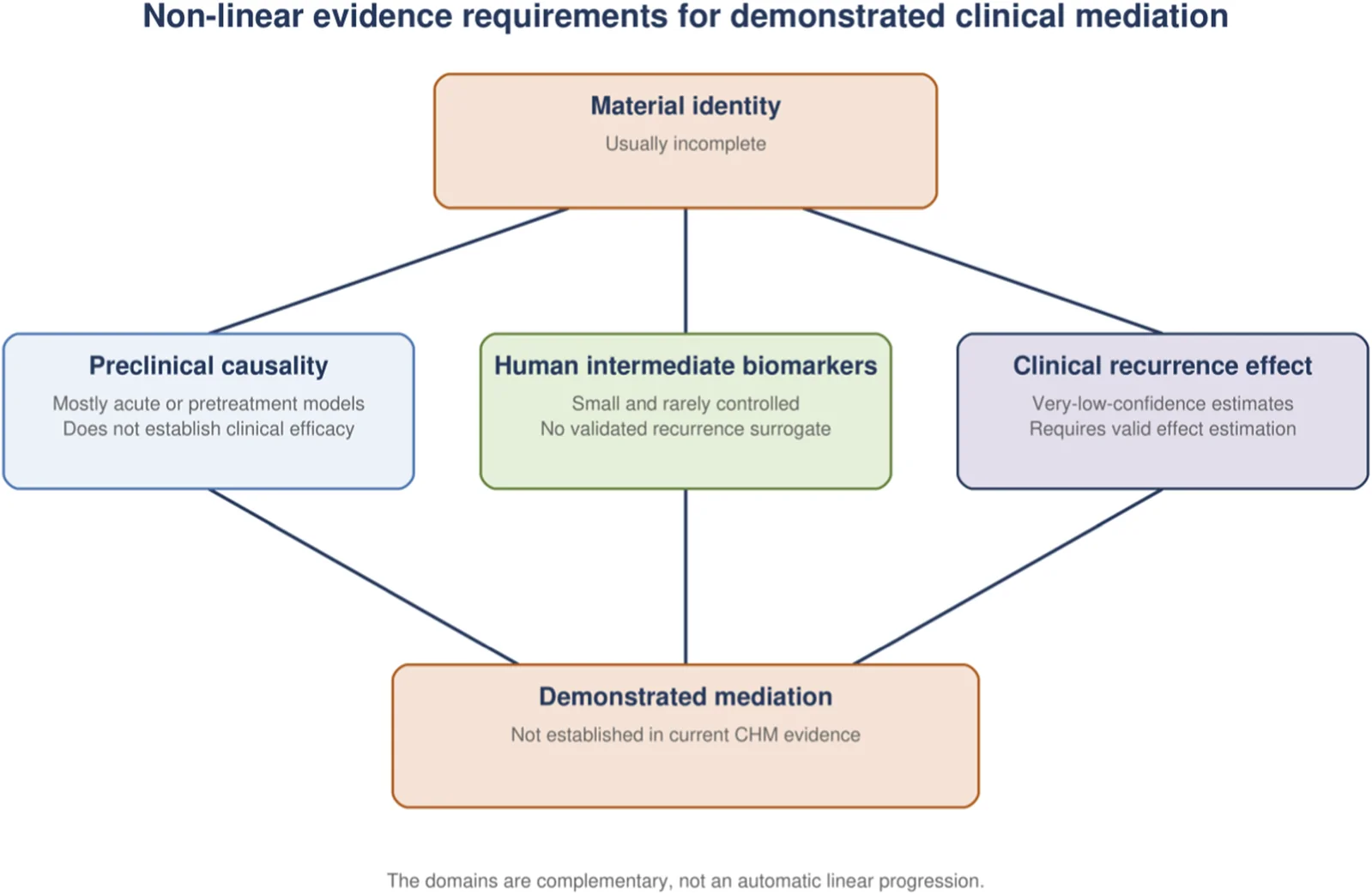
Search and evidence-mapping flow. Database and named-formula batches are displayed separately because they overlap and cannot be summed as unique records. Wanfang NF-B and NF-C carry-over rows and the invalid duplicate CNKI NF-C export were retained in the audit trail but were not treated as new attributable records. Following within-batch screening and cross-source reconciliation, 103 report records were linked into 96 clusters and classified as core (n = 10), supporting (n = 42), peripheral (n = 39), or exclude (n = 5). The figure describes the available search and evidence-mapping trail; it is not a systematic-review study-selection flow diagram.

Accordingly, current evidence does not show that CHM prevents recurrence by clearing intracellular UPEC, suppressing quiescent reservoirs, interrupting intestinal or vaginal transmission, restoring a protective urinary microbiome, or repairing urothelial barrier dysfunction. These remain testable hypotheses. Plausibility is not equivalent to clinical mediation.

### 6.2 Ethnopharmacological relevance, identity, and product characterization

Ethnopharmacological interpretation requires accurate identification of the intervention. Many clinical reports used traditional drug names without accepted botanical names, author citations, medicinal parts, processing, extraction yield, manufacturer, batch number, pharmacopoeial standard, chemical fingerprint, quantified markers, or voucher specimens. For example, comparative LC–MS/MS profiling detected significant differences in compound levels between two Astragalus taxa and among five plant organs, illustrating why botanical identity and medicinal part are important for reproducible characterization of herbal interventions (Li et al., 2019). Similar English names can also conceal different prescriptions, and individualized formulae vary between participants. Table 2 summarizes the extent to which major clinical interventions were reproducibly characterized. An expanded intervention-level matrix is provided in Supplementary Table S4.

**TABLE 2:** Characterization of major clinical CHM interventions. NR, not reported. Botanical species were not retrospectively inferred when source reports used ambiguous crude-drug names or omitted medicinal parts. The table records information in the study program and does not imply chemical or botanical equivalence across products.

Intervention	Type and composition reporting	Preparation/manufacturer	Authentication and chemistry	Main reproducibility implication
Modified Bazheng formula	Fixed granule formula; ingredients and doses reported	Formula granules; Jiangyin Tianjiang; batch 20100219	No voucher, study-batch fingerprint, or quantified markers	Clinically well specified relative to other studies, but chemical equivalence is unproven
Individualized CHM	Two eight-drug base prescriptions and doses reported, with symptom-based additions	Daily decoctions; active one-tenth-dose granule comparator produced by Tianjiang; treatment-product supplier/batches NR	Botanical authentication and chemical characterization NR	Participant-level ingredient frequencies and exposure are required; not one fixed product
Er Ding Er Xian/Zi Shen Qing Li	Two syndrome-selected eight-drug base prescriptions with doses; additions permitted; source English pharmacognostic names contain at least one crude-drug/source inconsistency	Daily water decoctions; treatment suppliers/batches NR; control used Tianjiang one-tenth-dose active formula granules	Study-batch species/parts, authentication, voucher, fingerprint, and quantitative markers NR	Base recipes are describable, but participant-level exposure and batch equivalence are unresolved
Sanjin tablets	Source reports name five crude drugs, with one report using “etc.” and one substituting the source-reported synonym Jingangci for Baqia	Tablets; Guilin Sanjin reported in 2009/2011 studies; manufacturer NR in 2008; all batches NR	Species, medicinal parts, study-batch authentication, fingerprint, and markers NR	A stable trade name does not establish botanical or batch equivalence across studies
Zhuolin granules	Thirteen crude drugs and doses reported	Hospital-provided preparation; Jiangyin Tianjiang; batch NR	Source species, medicinal parts, voucher, fingerprint, and markers NR	Recipe is reproducible at crude-drug level, but botanical and chemical equivalence is unresolved
Staged Yinqiao Bazheng → Zhibai Dihuang	Base ingredients, doses, modifications, and schedules reported	Water decoctions; supplier and batch NR	Source species, medicinal parts, authentication, voucher, fingerprint, and markers NR	Recipe is describable but botanical and chemical identity is not reproducible
Modified Gualou Qumai Wan	Five ingredients and doses reported	Conventional water decoction; manufacturer/batch NR	Source species and most medicinal parts NR; chemistry NR	A classical formula name does not ensure cross-study equivalence
Fuzheng Qingre Lishi	Only six ingredients named; full formula and doses unavailable	Unified hospital decoction; supplier and several crude-drug batches reported	Named drugs pharmacist-authenticated; fingerprint and markers NR	Partial composition prevents independent reproduction despite some sourcing data
Tailin/Tailin Fang II	Clinical Tailin names four crude drugs without doses; experimental Tailin Fang II names six drugs followed by “etc.”	Clinical manufacturer/batch NR; experimental drugs purchased from Shanghai WanShiCheng and pharmacy-identified; batch/voucher NR	Database-assisted UHPLC–MS annotation for the experimental preparation; authentic standards, quantitative markers, and batch replication NR	The incomplete formulas differ; clinical and experimental preparations must not be assumed equivalent

The staged Yinqiao Bazheng/Zhibai Dihuang reports illustrate the distinction between clinical recipe description and product reproducibility. The authors reported ingredients, doses, modifications, and decoction schedules, but not source species, medicinal parts, suppliers, batches, authentication, or chemical composition. Conversely, the modified Bazheng granule trial reported a manufacturer and batch, but no study-batch fingerprint or quantified marker compounds. Fuzheng Qingre Lishi reported some supplier and crude-drug batch information, yet the complete formula and doses were unavailable. Zhuolin reported a full formula and doses, but several source species, batch information, and chemical characterization were absent.

These gaps limit the level of inference but do not make every extractable clinical outcome unusable. When recurrence events and denominators are identifiable, findings can still contribute to descriptive synthesis and hypothesis generation. However, without verified botanical identity, medicinal parts, preparation, and batch composition, reports cannot support confident efficacy or safety claims for a reproducible product, establish equivalence across similarly named preparations, or enable faithful replication. When the administered material cannot be reconstructed, formulation-specific interpretation is not possible. Product characterization therefore determines the evidentiary use of a result rather than creating a binary choice between inclusion and exclusion.

The full-text audit also identified why finished-product naming alone is insufficient. The Yinhua Miyanling Tablet report supplied a finished-product lot, a complete ten-drug formula, and an HPLC similarity assessment across six batches but contained internal source-species or medicinal-part conflicts: Scutellariae Barbatae Herba was mapped to *Nelumbo nucifera* Gaertn., and Plantaginis Semen was described as stems and leaves (Zheng et al., 2025). These conflicts are retained in the appendix as source-reporting problems rather than silently corrected. By contrast, the experimental *P. capitata* extract had a traceable extract lot and validated quantitative analysis of four markers and is therefore a positive example of chemical characterization, although collection provenance, a voucher, extraction yield, and drug–extract ratio remained unreported (Zhou et al., 2025). Cynaroside is a purchased purified compound and was audited using compound-identity and purity fields rather than being treated as a botanical extract (Zheng et al., 2026).

Future reports should provide the complete prescription and dose; accepted botanical names and families; medicinal parts and processing; extraction and concentration procedures; dosage form; manufacturer and batch; applicable pharmacopoeial or quality standards; chromatographic or spectrometric fingerprints; quantified marker compounds; and voucher information where relevant. These elements are consistent with the ConPhyMP recommendations for reproducible characterization of medicinal plant extracts (Heinrich et al., 2022). Individualized prescriptions additionally require prespecified modification rules and participant-level ingredient frequencies. Reports of randomized CHM-formula trials should follow the CONSORT extension for Chinese herbal medicine formulae (Cheng et al., 2017). Where feasible, batch-linked certificates of analysis and supporting chromatographic or spectrometric data should accompany the publication, so readers can assess what was administered and whether another batch is comparable.

Where source reports permitted species-level identification, nomenclature was assessed against World Flora Online and Plants of the World Online (World Flora Online, 2026; Royal Botanic Gardens, Kew, 2026). The appendix distinguishes (i) the identity reported by the primary study, (ii) an externally mapped pharmacopoeial source where available, and (iii) verification of the actual study batch. We did not infer a study-batch species when a Chinese crude-drug name can correspond to multiple accepted sources, when the report omitted the medicinal part, or when the source itself was internally inconsistent. Missing species-level information is therefore recorded as a limitation rather than repaired retrospectively. Pharmacopoeial drug names were cross-checked against the supplied Chinese Pharmacopoeia 2025 where a monograph was available; regional standards were used for Yangkaikou and Hedyotidis Herba. These sources clarify external nomenclature only and do not retrospectively authenticate historical study batches. Jinshateng was not conflated with the spore drug Lygodii Spora, and unavailable monographs were recorded as not located. The full-text audit of selected experimental pharmacology studies is summarized in Table 3.

**TABLE 3:** Full-text audit of selected experimental pharmacology studies. Concentrations and doses are source-reported. The table assesses the experiment performed and does not imply clinical recurrence efficacy. *CAV1*, caveolin-1; CFU, colony-forming units; LVX, levofloxacin; NR, not reported; UPEC, uropathogenic *Escherichia coli*.

Test material	Identity and chemical characterization	Model and exposure	Comparator and main readouts	Interpretive boundary
Modified Zhibai Dihuang pill (Chen et al., 2023)	Thirteen crude drugs and doses reported; pharmacy-prepared water decoction; source batches, vouchers, and study-batch chemistry NR	ESBL-producing *E. coli* acute rat UTI; 20 g/kg/day for 14 days	LVX 0.025 g/kg/day; urine CFU, pathology, biofilm-associated, and inflammatory readouts	Acute single-infection model; no recurrence or relapse phase; product equivalence to clinical Zhibai Dihuang regimens unestablished
Tailin Fang II (Li et al., 2024)	Six named crude drugs followed by “etc.“; pharmacy identification reported; qualitative database-assisted UHPLC–MS annotation without complete formula or quantitative batch markers	CFT073 acute mouse UTI; 2.28 g/kg/day, with dosing initiated before inoculation	LVX comparator; bacterial burden, innate immune signaling, bladder injury, and inflammatory readouts	Incomplete formula and pretreatment design; experimental preparation cannot be assumed equivalent to clinical Tailin
Aqueous *P. capitata* extract (Zhou et al., 2025)	Finished extract lot 22136D; 78 constituents tentatively profiled; validated quantification of gallic acid, protocatechuic acid, quercitrin, and quercetin; raw-material provenance, voucher, extraction yield, and drug–extract ratio NR	UPEC bacterial assays (1–25 mg/mL), T24 cells (50–200 μg/mL), and acute rat UTI (0.32–1.28 g/kg/day); dosing began 2 days before infection	LVX; growth, motility, fimbriae/flagella, transcriptomics, extracellular vesicles, adhesion/invasion, urine and tissue outcomes	Comparatively strong chemical characterization, but single-challenge pretreatment model and no recurrence endpoint
Yinhua Miyanling Tablet (Zheng et al., 2025)	Ten-drug formula and finished-product lot 210,543 reported; six-batch HPLC similarity assessment; no quantified markers in this report; internal conflicts in source-species/medicinal-part mapping	Uncontrolled before–after study in 10 women for 28 days; acute CFT073 mouse UTI with 0.5–2 g/kg/day for 5 days	Human urine microscopy, serum cytokines, transcriptomics, and metabolomics; mouse LVX comparator, urine CFU, pathology, and TLR4/MyD88/p38 MAPK/NFκB readouts	Human intermediate biomarkers plus acute mouse plausibility; no comparative recurrence outcome, clinical mediation, or fully reliable botanical identity
Cynaroside (Zheng et al., 2026)	Purified compound, ≥98%, commercial supplier; lot and independent analytical identity confirmation NR; ConPhyMP botanical-extract fields not directly applicable	UPEC assays (commonly 125–500 μg/mL), 5,637 cells (10–40 μM), and acute rat UTI (10–40 mg/kg/day); dosing began 2 days before infection	LVX; virulence, motility, biofilm, adhesion/invasion, barrier, and cytokine measures; *CAV1* knockdown and rescue; urine CFU and pathology	Stronger cell-level causal evidence for *CAV1*, but a single metabolite is not a polyherbal preparation, and acute pretreatment does not establish recurrence prevention

### 6.3 Clinical and regulatory implications

The evidence reviewed here does not support replacing guideline-based evaluation or prevention of recurrent cystitis with CHM, nor does it support recommending CHM as a class. Clinicians who encounter patients already using a botanical preparation should first establish whether current symptoms are compatible with localized bacterial cystitis, obtain urine testing when clinically indicated, and assess for systemic infection, pregnancy, urinary tract abnormality, renal impairment, or another condition requiring conventional evaluation. Any discussion of adjunctive CHM should be product-specific and should address the exact formula, manufacturer and batch, treatment phase, concurrent antimicrobials or other medicines, allergy history, and the possibility that a reported absence of adverse events reflects incomplete ascertainment rather than demonstrated safety. Preparations should not delay antimicrobial treatment when it is clinically required.

The reviewed interventions cannot be assumed to be interchangeable across hospitals, manufacturers, countries, or regulatory categories. A formula name may refer to a commercial finished product, hospital preparation, individualized decoction, or experimental extract with different source drugs, processing, dose, chemistry, and quality controls. Regulatory pathways likewise distinguish investigational botanical drugs, authorized or registered herbal medicinal products, traditional-use products, and other marketed categories, and documentation attached to one category does not automatically transfer to another (U.S. Food and Drug Administration, 2016; European Medicines Agency, 2026). Cross-market extrapolation therefore requires batch traceability, applicable pharmacopoeial standards, contaminant and stability testing, and evidence that the tested product is sufficiently comparable to the product used in practice. When botanical identity or batch-linked composition is not reported, this comparability cannot be established, and findings should not be transferred to similarly named products.

The one-tenth-dose herbal preparations described as “placebos” in the 2017 and 2024 trials raise a distinct control-design concern. Dose reduction alone does not establish pharmacological inactivity; residual activity could alter the estimated treatment contrast or adverse-event profile, although its direction and magnitude cannot be inferred from the reports. As one illustrative US regulatory standard, FDA guidance advises that botanical materials used to mask a placebo should have no known pharmacological activity (U.S. Food and Drug Administration, 2016). A low-dose botanical with plausible residual activity should therefore be prespecified and reported as a potentially active comparator, not assumed inert solely because its dose is reduced. If that activity is not characterized or disclosed, the control may also complicate the interpretation of blinding and participants’ understanding of what they are taking. Future protocols, registries, ethics submissions, and consent materials should state the comparator’s composition, dose, known or plausible activity, risks, and rationale. This approach is consistent with the Declaration of Helsinki’s requirements for justified protocols, risk assessment, informed consent, and carefully justified placebo use (World Medical Association, 2024). The available reports do not establish whether either trial breached its applicable requirements.

### 6.4 Research priorities

A decisive clinical trial should evaluate a botanically and chemically characterized product during remission, use a standard definition of recurrent symptomatic culture-confirmed cystitis, and analyze all randomized participants. Comparator selection should distinguish monotherapy from adjunctive treatment and use a matched placebo with no known pharmacological activity when placebo control is appropriate. A low-dose botanical with plausible residual activity should instead be prespecified and reported as a potentially active comparator. Recurrence should be reported as participant events, episode counts, and time-to-event outcomes, with prespecified handling of relapse, reinfection, attrition, antimicrobial rescue, adherence, and adverse events. Allocation concealment, blinded endpoint adjudication, trial registration, protocol–publication concordance, and public statistical analysis plans are essential.

Mechanistic sampling should be longitudinal and anatomically explicit. Stool, vaginal specimens, catheterized urine, and, where ethically justified, urothelial material should be collected before treatment, during remission, and at recurrence. Whole-genome sequencing should distinguish same-strain persistence from reinfection. Prespecified mediation models should test whether changes in pathogen burden, strain carriage, microbial communities, mucosal immunity, or barrier function explain clinical effects. Parallel recurrent-infection models should incorporate antibiotic treatment, relapse phases, causal perturbation or rescue, and blinded assessment rather than relying on pretreatment protection against a single acute challenge.

### 6.5 Strengths and limitations of this review

This review used a structured search, a frozen evidence inventory, and report-level linkage, but it was not prospectively designed as a systematic review. The initial author-assembled seed set lacked recoverable database search records; subsequent database batches and reconciliation steps are described in Section 2.2 and Supplementary Table S3. SinoMed/CBM could not be searched because institutional access was unavailable, and several Chinese reports remained available only as abstracts, metadata, or secondary descriptions. The directness hierarchy was designed for critical interpretation rather than formal risk-of-bias or GRADE assessment. The estimated independent-study range reflects unresolved title-level records and one internally unreliable report. Full botanical and chemical verification was possible for only a subset of interventions.

The review’s principal strengths are report-level linkage, explicit separation of directness from quality, targeted audit of decisive full texts, recalculation of incompatible significance claims, preservation of recurrence denominators, and separation of clinical outcomes from biomarker and mechanistic plausibility. These safeguards reduce the risk of an overly favorable narrative but cannot correct weaknesses in primary reporting. Our targeted recalculations identified significance claims incompatible with reported recurrence counts in selected studies, including the staged-regimen report discussed in Section 3.6. Because this audit was targeted rather than representative, it cannot estimate the prevalence of statistical reporting errors across the field. The observed discrepancies nevertheless warrant stricter editorial and statistical checks. Primary publications should make recurrence events, analysis denominators, statistical test choices, and exact P-values auditable where feasible; editors and statistical reviewers should verify claims that materially affect clinical conclusions.

## 7 Conclusion

Selected CHM products and individualized prescriptions have generated preliminary, formulation-specific signals in women with recurrent or chronic UTI, particularly as adjuncts after control of acute infection. The evidence is heterogeneous and methodologically fragile and does not justify a class-wide efficacy claim. Poor product characterization does not make every extractable outcome unusable: identifiable recurrence counts and denominators may still inform descriptive synthesis and hypothesis generation. However, without verifiable botanical identity, preparation, and batch composition, reports cannot support confident product-specific efficacy or safety claims, establish equivalence across products, or enable faithful replication. Experimental studies support plausible antimicrobial, anti-adhesion, anti-inflammatory, immune, and urothelial actions, but most models do not represent clinical recurrence. Direct evidence linking CHM benefit to persistent uropathogens, gut–bladder or vaginal transmission, local microbial ecology, mucosal immunity, or urothelial barrier repair is absent. Progress requires complete, reproducible product characterization, rigorous intention-to-treat recurrence trials, strain-resolved longitudinal sampling, and prespecified mediation analyses.
